# Central Venous Pressure Measurement Is Associated With Improved Outcomes in Patients With or at Risk for Acute Respiratory Distress Syndrome: An Analysis of the Medical Information Mart for Intensive Care IV Database

**DOI:** 10.3389/fmed.2022.858838

**Published:** 2022-03-28

**Authors:** Rui Tang, Junnan Peng, Daoxin Wang

**Affiliations:** Department of Respiratory and Critical Care Medicine, The Second Affiliated Hospital of Chongqing Medical University, Chongqing, China

**Keywords:** CVP, ARDS, 28-day mortality, critical care, lactate

## Abstract

**Background:**

Central venous pressure (CVP) monitoring is widely used in the intensive care unit (ICU). However, the formal utility of CVP measurement to altering patient outcomes among ICU patients with or at risk for acute respiratory distress syndrome (ARDS) has never been investigated. Our study aimed to explore the association of CVP measurement with 28-day mortality specifically in that population.

**Methods:**

This study was based on the Medical Information Mart for Intensive Care IV (MIMIC-IV) database. Patients were divided into CVP and no CVP groups according to whether they had CVP measurement within 24 h of admission to the ICU. The primary outcome was 28-day mortality. Multivariate regression was used to elucidate the association between CVP measurement and 28-day mortality, and propensity score matching (PSM) and propensity score-based overlap weighting (OW) were employed to verify the stability of our results.

**Results:**

A total of 10,198 patients with or at risk for ARDS were included in our study, of which 4,647 patients (45.6%) belonged to the CVP group. Multivariate logistic regression showed that the early measurement of CVP was independently associated with lower 28-day mortality (OR: 0.49; 95% CI: 0.42–0.57; *p* < 0.001). This association remained robust after PSM and OW (both *p* < 0.001). Patients in the CVP group had shorter ICU stay, lower in-hospital mortality, more fluid on day 1 and higher clearance of blood lactate than those in the no CVP group.

**Conclusion:**

Early CVP measurement is associated with an improvement in 28-day mortality among a general population of critically ill patients with or at risk for ARDS.

## Introduction

Acute respiratory distress syndrome (ARDS) remains a major challenge in the intensive care unit (ICU), responsible for significant morbidity and mortality (1). The pathophysiologic hallmark of ARDS is the disruption of microvascular endothelium and alveolar epithelium, leading to increased permeability of the alveolar-capillary barrier and subsequent pulmonary edema (2). Previous studies have found that over 60% of ARDS patients present hemodynamic instability (3, 4), indicating the importance of fluid management in ARDS. However, fluid administration is a double-edged sword: although it supports cardiac output and vital tissue perfusion, the increased hydrostatic pressure can result in lung edema formation (5). Thus, how to walk this tightrope has become one of the most flourishing research topics in recent decades.

Central venous pressure (CVP) has been employed as an indicator of intravascular volume and cardiac preload for over 60 years (6). In a survey about perioperative hemodynamic monitoring in high-risk surgical patients, 73% of American and 84% of European anesthetists reported that they used the CVP to guide fluid management (7). CVP has long been considered as a component in the assessment of adequate fluid resuscitation in patients with septic shock (8), despite recent challenges to its reliability and validity (9). The Fluid and Catheter Treatment Trial (FACTT) also indicated that initial CVP could predict fluid responsiveness in patients with ARDS (10). While CVP measurement has been commonly used in the ICU to aid in clinical decision-making, the implications for patient outcomes have barely been reported. In a randomized controlled trial of patients undergoing proximal femoral fracture repair under general anesthesia, CVP measurement and oesophageal Doppler ultrasonography shortened time to being medically fit for discharge (11). A retrospective study conducted by Chen et al. found that CVP measurement during ICU stay could decrease the risk-adjusted 28-day mortality in septic patients (12). Another retrospective study demonstrated that the usage of CVP and echocardiography was associated with lower mortality in patients with acute gastrointestinal hemorrhage (13). To date, the formal utility of CVP measurement in predicting mortality is still unclear in patients with or at risk for ARDS.

In the present study, we aimed at elucidating the effect of early CVP measurement on 28-day mortality in patients with or at risk for ARDS. We hypothesized that the early usage of CVP measurement might decrease the 28-day mortality because it may help to expedite hemodynamic stabilization.

## Methods

### Study Design

This study is reported according to the Reporting of Studies Conducted using Observational Routinely Collected Health Data (RECORD) statement (14). We conducted this retrospective study based on a large critical care database named Medical Information Mart for Intensive Care IV (MIMIC-IV). The description of MIMIC-IV is available elsewhere (15). Briefly, the MIMIC-IV database contains comprehensive and high-quality clinical data of the patients admitted to the ICU at the Beth Israel Deaconess Medical Center (Boston, Massachusetts) between 2008 and 2019. This database was approved by the Institutional Review Boards of the Massachusetts Institute of Technology. We have completed the National Institutes of Health Web-based training course and the Protecting Human Research Participants examination (No. 9555299) to gain access to the database. Given the retrospective nature of this study and the use of anonymized data, the approval of our institution and informed consent were exempted.

### Selection of Patients

We screened all the patients in the database, and all patients who met the following criteria were included: (1) age older than 16 years, (2) length of ICU stay more than 24 h, (3) with or at risk for ARDS. The current diagnostic criteria for ARDS follow the Berlin definition (16), which included: (1) acute onset of respiratory symptoms; (2) presence of bilateral infiltrates on chest radiograph; (3) arterial oxygen partial pressure (PaO_2_)/fraction of inspired oxygen (FiO_2_) <300 mmHg with a minimum requirement for positive end-expiratory pressure (PEEP) ≥ 5 cmH_2_O; (4) absence of heart failure. On ICU admission, patients who met all four Berlin criteria were considered to have a clinical diagnosis of ARDS. Moreover, our primary purpose was to explore the effect of early use (<1 day) of CVP on 28-day mortality, but the chest imaging was sometimes delayed or missed for many ICU patients in the MIMIC-IV database. Therefore, we also used the remaining three Berlin criteria for diagnostic assessment and considered patients as at risk of ARDS. No other exclusion criteria were applied. In the case of multiple ICU admissions, we only used the data of each patient’s first ICU admission. The patients who had their CVP measurement within 24 h after ICU admission were categorized as the CVP group, with the remaining patients making up the no CVP group. The disease severity was classified into mild (200 mmHg < PaO_2_/FiO_2_ ratio ≤ 300 mmHg), moderate (100 mmHg < PaO_2_/FiO_2_ ratio ≤ 200 mmHg), or severe (PaO_2_/FiO_2_ ratio ≤ 100 mmHg) according to the Berlin criteria.

### Variable Extraction

We used the Structured Query Language with Navicat Premium (version 15.0.21) to extract the data. The primary exposure of interest was whether a patient underwent CVP measurement. The patients who had CVP measurement within 24 h of admission to the ICU were categorized as the CVP group, with the remaining patients forming the no CVP group. The time to initial CVP measurement and the initial level of CVP were also recorded.

Baseline characteristics within the first 24 h after ICU admission were collected, including age, sex, body mass index (BMI), ethnicity, admission type, admission period, first care unit, risk factors of ARDS, the severity of illness and organ dysfunction as measured by acute physiology score III (APS III), oxford acute severity of illness score (OASIS) and logistic organ dysfunction system (LODS). Vital signs included temperature, heart rate, respiratory rate and mean arterial pressure (MAP), were also extracted. The PaO_2_/FiO_2_ and positive terminal expiratory pressure (PEEP) were collected at the time of diagnosis. The laboratory variables, including white blood cell, hemoglobin, platelet, bicarbonate, blood urea nitrogen, creatinine, lactate, glucose, sodium and potassium were collected within 24 h of ICU admission. If a variable was recorded more than once in the first 24 h, we used the value at the first measurement. In our study, all missing values were imputed using a miss forest imputation, and the details about missing values can be found in Supplementary Table 1.

All comorbidities were identified by the International Classification of Diseases (ICD)-9 or ICD-10 codes in the MIMIC-IV database, including hypertension, coronary atherosclerosis, diabetes, chronic obstructive pulmonary disease (COPD) and tumor. The Charlson comorbidity score was used for comorbidity assessment.

### Outcomes

The primary outcome in the present study was 28-day mortality, which was defined as the occurrence of death at 28 days from the date of ICU admission. Secondary outcomes included in-hospital mortality, length of ICU stay, the incidence of acute kidney injury within 7 days after ICU admission, the volumes (L) of intravenous fluid (IVF) input and balance in the first, second and third days in the ICU, clearance of lactate [calculated as (maximum lactate on day 3- maximum lactate on day 1)/maximum lactate on day 1 × 100%] (17), the use of ventilation and continuous renal replacement therapy during hospitalization (yes/no). AKI was diagnosed based on the Kidney Disease Improving Global Outcomes (KDIGO) criteria (18).

### Statistical Analysis

We used R statistical software (version 3.6.1), GraphPad Prizm (version 8.0, San Diego, CA, United States) or SPSS software (version 26.0, IBM, United States) to perform statistical analysis and create pictures. Descriptive statistics were performed to characterize the study patients. Normally distributed continuous variables were presented as mean ± standard deviation, non-normally distributed continuous variables as median (interquartile range), and categorical variables as frequencies (percentages). For between-group comparisons, we used Student’s *t*-test, Mann-Whitney *U* test and Chi-square test for normally distributed continuous variables, non-normally distributed continuous variables, and categorical variables, respectively.

Multivariate regression analyses served to evaluate the relationship between CVP measurement and 28-day mortality. The adjusted variables included age, gender, BMI, ethnicity, admission period, first care unit, Charlson comorbidity score, risk factors of ARDS, APS III, OASIS, LODS, PaO_2_/FiO_2_ at diagnosis, WBC, creatinine and lactate. We selected confounders based on their possible associations with the outcomes or a *p*-value less than 0.1 in univariable analyses. Variables for final inclusion were carefully chosen, given the number of events available, to ensure parsimony of the model.

We further used propensity score matching (PSM) and propensity score-based overlap weighting (OW) to balance the covariables and ensure the robustness of our results. The PSM method is usually applied to reduce or eliminate the confounding effects when using observational data (19). In our study, the estimation algorithm of propensity score was logistic regression and the matching algorithm was one-to-one matching with a caliper width of 0.10. The OW is an extensive propensity score method that mimics attributes of a randomized clinical trial (20). We constructed the OW model using the estimated propensity scores as weights. After PSM and OW, the standardized mean differences (SMDs) were calculated to illustrate the effect sizes. We considered an SMD of less than 0.1 as acceptable. Then, logistic regression was conducted on the matched cohort and weighted cohorts to calculate the odds ratios (ORs) for 28-day mortality. In the PSM cohort, we used Student’s *t*-test or Chi-square test to compare secondary outcomes, as appropriate.

All tests were two-sided with significance at *p* < 0.05.

### Sensitivity Analysis

We performed multiple sensitivity analyses to evaluate the impact of study design variability on our results. The initial CVP level may influence the effect of CVP measurement (12). Therefore, we conducted sensitivity analyses comparing patients of initial CVP level was below 8 mmHg or above 15 mmHg with patients in the no CVP group. Moreover, we performed subgroup analyses by age, gender, first care unit, Berlin classification and LODS score for our primary outcome.

## Results

### Baseline Characteristics

**FIGURE 1:**
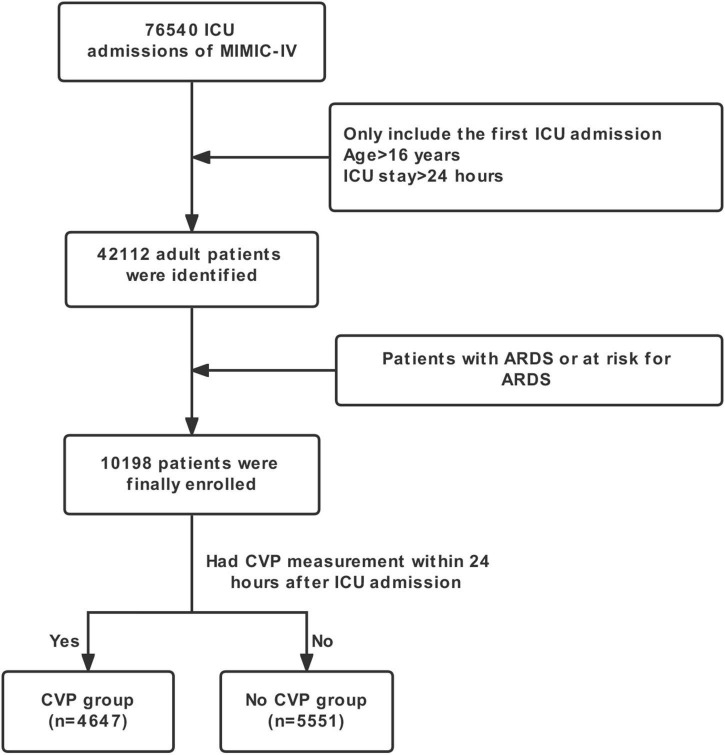
Flow chart of this study.

After reviewing the records of 76,540 ICU patients from the MIMIC-IV database, we included 10,198 patients with or at risk for ARDS in the present study. A flow diagram of the selection is shown in Figure 1. Among the study cohort, CVP measurement was performed for 48.6% of patients during the first 24 h after their ICU admission. Characteristics of CVP and no CVP groups are outlined in Table 1. In general, patients in the CVP group were older, had more male, Caucasian, and lower severity scores on admission: APS III 50.79 ± 29.46 vs. 59.05 ± 27.69, LODS 5 (3–8) vs. 6 (4–9), OASIS 34.56 ± 9.67 vs. 38.23 ± 8.43.

**TABLE 1:** Baseline characteristics between the original cohort and matched cohort.

Covariates	Original cohort			Matched cohort		
	No-CVP group (*n* = 5,551)	CVP group (*n* = 4,647)	SMD	No-CVP group (*n* = 1,779)	CVP group (*n* = 1,779)	SMD
Age (years)	60.47 ± 17.27	65.49 ± 13.71	0.322	62.11 ± 16.59	61.80 ± 15.80	0.019
Man (n, %)	3,173 (57.2)	3,144 (67.7)	0.218	1,078 (60.6)	1,072 (60.3)	0.007
BMI (kg/m^2^)	29.08 ± 6.88	29.42 ± 6.21	0.052	29.63 ± 7.37	29.32 ± 6.78	0.044
Ethnicity, n (%)			0.272			0.015
White	3,266 (58.8)	3,331 (71.7)		1,163 (65.4)	1,176 (66.1)	
Non-white	2,285 (41.2)	1,316 (28.3)		616 (34.6)	603 (33.9)	
Admission type, n (%)			0.153			0.011
Emergency	3,752 (67.6)	2,800 (60.3)		1,208 (67.9)	1,199 (67.4)	
Non-emergency	1,799 (32.4)	1,847 (39.7)		571 (32.1)	580 (32.6)	
Admission period, n (%)			0.221			0.038
Before 2013	2,771 (49.9)	2,828 (60.9)		1,109 (62.3)	1,142 (64.2)	
After 2013	2,780 (50.1)	1,819 (39.1)		670 (37.7)	637 (35.8)	
First care unit, n (%)			1.659			0.014
CVICU/CCU	458 (8.3)	3,282 (70.6)		453 (25.5)	448 (25.2)	
MICU	2,366 (42.6)	667 (14.6)		661 (37.2)	654 (36.8)	
SICU/TSICU	2,727 (49.1)	688 (14.8)		665 (37.4)	677 (38.1)	
**Comorbidity, n (%)**						
Hypertension	2,554 (46.0)	2,678 (57.6)	0.234	827 (46.5)	805 (45.3)	0.025
Coronary atherosclerosis	291 (5.2)	1,418 (30.5)	0.699	178 (10.0)	171 (9.6)	0.013
Diabetes	1,158 (20.9)	1,198 (25.8)	0.117	412 (23.2)	411 (23.1)	0.001
COPD	437 (7.9)	160 (3.4)	0.193	88 (4.9)	93 (5.2)	0.013
Tumor	1,108 (20.0)	509 (11.0)	0.251	339 (19.1)	354 (19.9)	0.021
Charlson score	5 (3–7)	4 (3–6)	0.046	5 (3–7)	5 (3–7)	0.001
**Risk factor, n (%)**						
Pneumonia	941 (17.0)	289 (6.2)	0.34	221 (12.4)	233 (13.1)	0.02
Sepsis	1,200 (21.6)	674 (14.5)	0.186	574 (32.3)	588 (33.1)	0.017
Trauma	592 (10.7)	398 (8.6)	0.071	220 (12.4)	224 (12.6)	0.007
Others	309 (5.6)	164 (3.5)	0.098	131 (7.4)	138 (7.8)	0.015
**Severity scores**						
APS III	59.05 ± 27.69	50.79 ± 29.46	0.289	63.64 ± 30.26	65.53 ± 32.40	0.06
LODS	6 (4–9)	5 (3–8)	0.215	7 (4–10)	7 (4–10)	0.053
OASIS	38.23 ± 8.43	34.56 ± 9.67	0.405	38.59 ± 8.94	38.98 ± 10.05	0.041
**Baseline vital data**						
Temperature (^°^C)	36.67 ± 2.62	36.30 ± 2.71	0.139	36.48 ± 3.68	36.51 ± 3.38	0.007
HR (beats/min)	91.81 ± 21.42	85.51 ± 17.77	0.32	93.37 ± 21.94	93.67 ± 21.89	0.014
RR (times/min)	20.49 ± 6.51	16.88 ± 5.88	0.581	19.63 ± 6.18	19.84 ± 6.92	0.032
MAP (mmHg)	82.19 ± 33.68	77.96 ± 27.21	0.138	78.73 ± 31.71	78.74 ± 39.70	0.001
PaO_2_/FiO_2_ at diagnosis (mmHg)	176.67 ± 75.98	196.21 ± 71.83	0.264	178.58 ± 76.58	177.62 ± 77.50	0.012
PEEP at diagnosis (cmH_2_O)	5 (5–7)	5 (5–5.6)	0.13	5 (5–8)	5 (5–8)	0.045
**Laboratory findings**						
WBC (k/μL)	13.23 ± 9.39	13.40 ± 7.55	0.021	13.90 ± 13.34	13.91 ± 9.11	0.001
Hemoglobin (g/L)	11.01 ± 2.31	10.03 ± 1.99	0.452	10.54 ± 2.32	10.61 ± 2.20	0.033
Platelet (k/μL)	212.03 ± 114.21	167.66 ± 88.40	0.433	192.81 ± 110.60	194.07 ± 119.45	0.011
Bicarbonate (mEq/L)	22.42 ± 5.04	22.37 ± 3.64	0.011	21.39 ± 5.22	21.42 ± 4.66	0.008
Bun (mg/dL)	18 (12–28)	17 (13–22)	0.16	20 (13–32)	19 (14–32)	0.02
Creatinine (mg/dL)	0.9 (0.7–1.3)	0.9 (0.7–1.2)	0.092	1 (0.7–1.5)	1 (0.7–1.6)	0.009
Lactate (mmol/L)	1.7 (1.2–2.7)	2.1 (1.5–3.0)	0.128	1.8 (1.3–3.2)	2.2 (1.4–3.5)	0.041
Glucose (mg/dL)	150.21 ± 74.94	137.35 ± 62.81	0.186	156.46 ± 84.65	157.13 ± 83.57	0.008
Sodium (mEq/L)	138.82 ± 5.55	139.11 ± 4.08	0.059	138.74 ± 5.70	138.88 ± 5.25	0.024
potassium (mEq/L)	4.18 ± 0.78	4.29 ± 0.65	0.149	4.26 ± 0.84	4.26 ± 0.77	0.002

*Data were presented as mean ± standard deviation or median (interquartile range) or numbers (percentages).*

*CVICU, Cardiac Vascular Intensive Care Unit; CCU, Coronary Care Unit; MICU, Medical Intensive Care Unit; SICU, Surgical Intensive Care Unit; TSICU, Trauma Surgical Intensive Care Unit; COPD, Chronic obstructive pulmonary disease; APS III, Acute Physiology Score III; LODS, Logistic organ dysfunction system; OASIS, Oxford Acute Severity of Illness Score; HR, Heart Rate; RR, Respiratory rate; MAP, mean arterial pressure; WBC white blood cell.*

### Primary Outcome and Sensitivity Studies

**FIGURE 2:**
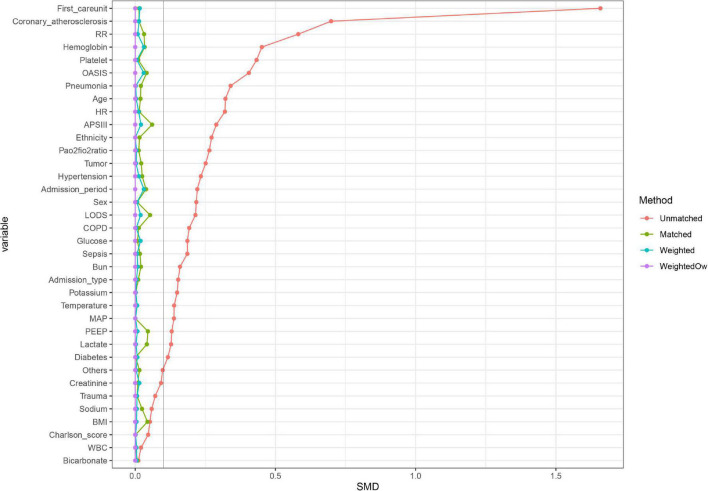
A graph showing the covariate balance of the matching balance effect. Propensity score MW, Propensity score matching weight; Propensity score OW, Propensity score overlap weight.

**FIGURE 3:**
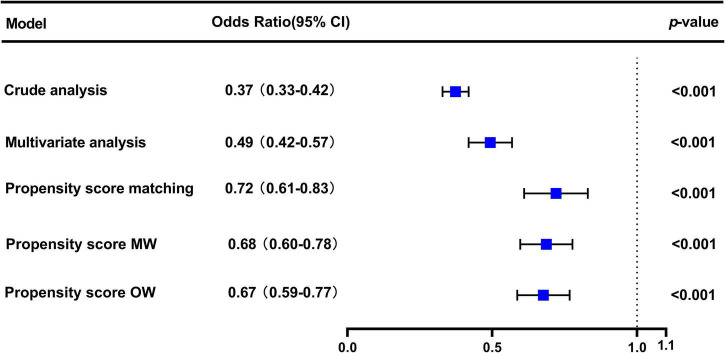
Association between early CVP measurement and 28-day mortality. The odds ratios and 95% confidence intervals (error bars) in the cohorts were calculated dependent on the method of covariate adjustment. Propensity score MW, Propensity score matching weight; Propensity score OW, Propensity score overlap weight.

**FIGURE 4:**
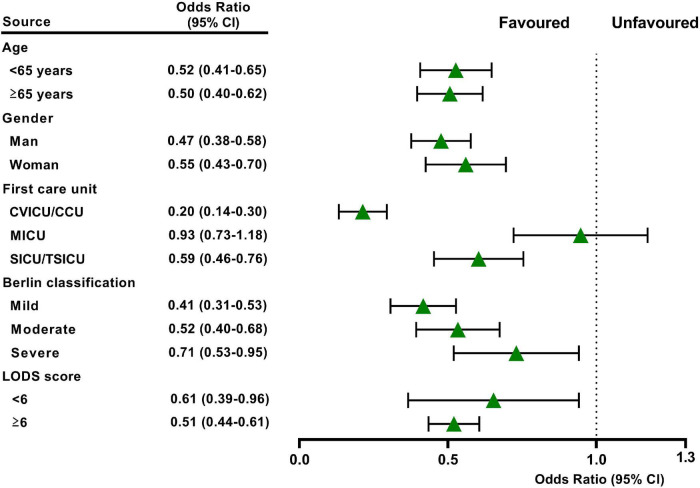
Subgroup analysis showed the relationship between early CVP use and 28-day mortality in different subgroups. The model was adjusted by age, gender, body mass index, race, first care unit, Charlson score, pneumonia, sepsis, trauma, others, early CVP use, APS III score, LODs score, OASIS score, ARDS severity, WBC, creatinine, and lactate.

**FIGURE 5:**
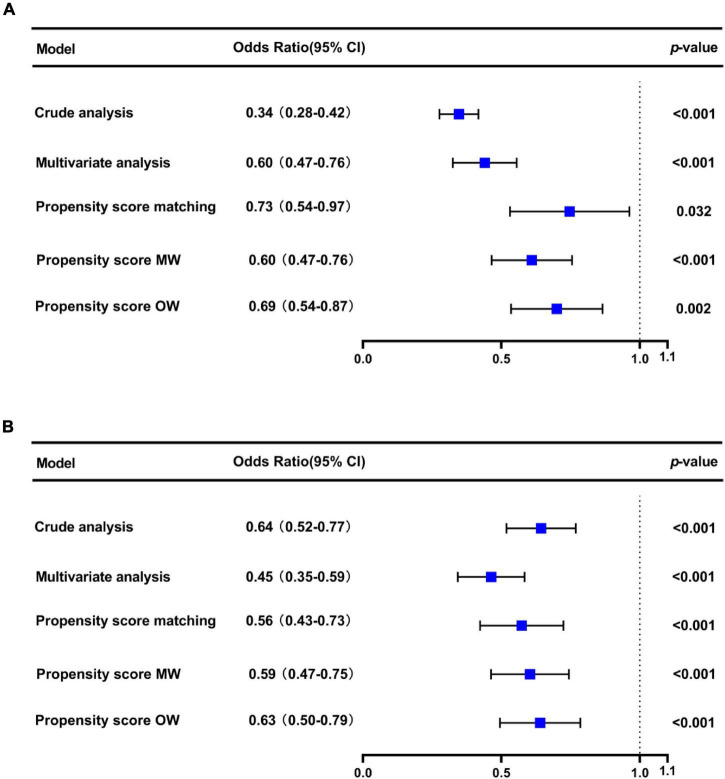
Sensitivity analysis showed that the prognosis of patients with initial CVP < 8 mmHg **(A)** and CVP > 15 mmHg **(B)**.

In the original cohort, the 28-day mortality in the CVP group was significantly lower than that in the no CVP group (10.22 vs. 23.40%, *p* < 0.001). The same result was found in the Kaplan-Meier analysis (Supplementary Figure 1). To eliminate the possible effect of confounders, we first used multivariate logistic regression. The results showed that the usage of CVP was independently associated with lower 28-day mortality (OR: 0.49; 95% CI: 0.42–0.57; *p* < 0001). Then, we used the PSM and OW methods to verify our findings. The overall between-group characteristics were well-balanced after PSM and OW (Table 1 and Figure 2), and the association between CVP measurement and 28-day mortality remained robust (Figure 3). Subgroup analyses were also performed according to age, gender, first care unit, Berlin classification and LODS score. As shown in Figure 4, the beneficial effect of CVP measurement on 28-day mortality stably existed except for patients of MICU. Finally, we conducted two sensitivity analyses with different initial CVP value, and similar results were observed (Figure 5).

### Secondary Outcomes

Table 2 shows the differences in secondary outcomes between the CVP and no CVP groups. Patients in the CVP group had a shorter ICU stay and lower in-hospital mortality, but the incidence of AKI was comparable between the two groups. Some potential factors that might account for the beneficial effects of CVP measurement were also investigated. When compared to the no CVP group, the amount of fluid input and balance on day 1 were both significantly higher in the CVP group (both *p* < 0.001). Regarding the lactate levels, we found the clearance of serum lactate from day 1 to day 3 was higher in the CVP group than in the no CVP group (33 vs. 19%, *p* < 0.001).

**TABLE 2:** Secondary outcomes analysis with propensity score matched cohorts.

Variables	no-CVP group (*n* = 1,779)	CVP group (*n* = 1,779)	*p*
In-hospital mortality, n (%)	496 (27.9)	391 (22.0)	<0.001
28-day mortality, n (%)	486 (27.3)	377 (21.2)	<0.001
AKI in 7 days, n (%)	1,508 (84.8)	1,511 (84.9)	0.888
Length of ICU stay, days	4.58 (2.39–8.99)	4 (2.02–8.71)	0.001
Input day 1 (L), median (IQR)	5.56 (3.25–9.05)	8.55 (5.91–12.56)	<0.001
Input day 2 (L), median (IQR)	3.12 (1.20–5.91)	3.36 (1.10–6.80)	0.165
Input day 3 (L), median (IQR)	2.10 (0.7–4.61)	1.76 (0.50–4.72)	<0.001
Fluidbalance day 1, median (IQR)	3.11 (0.82–6.65)	5.62 (2.66–9.67)	<0.001
Fluidbalance day 2, median (IQR)	1.31 (-0.29 to 4.06)	1.31 (-0.35 to 4.62)	0.359
Fluidbalance day 3, median (IQR)	0.18 (-0.56 to 2.79)	-0.22 (-0.64 to 2.57)	0.005
Ventilation, n (%)	1,700 (95.6)	1,720 (96.7)	0.082
CRRT, n (%)	192 (10.8)	189 (10.6)	0.871
Clearance of lactate,%	19 (-2 to 44)	33 (12–55)	<0.001

*AKI, acute kidney injury; CRRT, Continuous renal replacement therapy.*

## Discussion

To our knowledge, this is the first study to explore the effects of CVP measurement on the short-term outcomes in ICU patients with or at risk for ARDS. Our results revealed that CVP measurement was significantly associated with lower 28-day mortality, shorter ICU stay, and lower in-hospital mortality. Moreover, patients in the CVP group received more fluid on day 1 and had a higher clearance of lactate than those in the no CVP group, but the incidence of AKI was similar between the two groups.

Acute respiratory distress syndrome is the frequent reason for ICU admission and sometimes associated with hemodynamic instability (1, 3, 4). As the high early mortality of ARDS, timely intervention may be critical to improve the patient prognosis and reduce mortality (21). CVP is routinely used in the hemodynamic assessment of critically ill patients (5). However, the association of CVP measurement with 28-day mortality in patients with or at risk for ARDS has never been formally examined. Identifying the clinical application and value of interventions is enormously essential. At times, an intervention, which brings benefit to specific patient populations, may expose other populations to harm. Therefore, some researchers advocate using big data to measure the impact of an intervention on patient-centered outcomes for a specific population (12, 22), and guide clinical strategies.

In our study, patients in the CVP group had lower severity of illness scores and fewer comorbid conditions, indicating less impairment of overall function. Thus, the 28-day mortality was significantly lower in the CVP group according to univariate and Kaplan-Meier analysis was not unexpected. To greatly remove bias induced by confounding factors, we then used the multivariate logistic regression, PSM and OW methods. All of the above analysis results supported the beneficial effect of CVP measurement on 28-day mortality for patients with or at risk for ARDS. Li et al. have demonstrated that the initial CVP level correlated with clinical outcome and treatment duration to all patients in critical care settings (23). In ARDS, Semler et al. found that initial CVP would modify the effect of fluid management on 60-day mortality (24). In this study, we compared the primary outcome between patients of initial CVP level was below 8 mmHg or above 15 mmHg with those in the no CVP group, separately. The results were still stable in the two analyses, highlighting the necessity of CVP measurement for ARDS, irrespective of its initial value. Furthermore, although the central venous catheter is frequently used in the ICU, its utilization may vary from different units (25). Age, sex, oxygenation and comorbidity status may also be confounders of study on CVP in ARDS (10, 24). Therefore, we adjusted these confounders and did a further subgroup analysis. These results remained robust except for patients of MICU. This is perhaps because surgical ARDS patients who are often younger and with lower comorbidities compared to medical ARDS patients, who develop ARDS in the context of multiple organ failure and thus might seem to require a different fluid management approach. As has been proven by many studies, medical patients have worse prognosis than surgical patients in the ICU (26–29).

Previous study has well-proved that the beneficial effects of CVP were mediated *via* the reduction in serum lactate (12). In this study, we also found that the clearance of serum lactate was significantly higher in the CVP group. Blood lactate is considered a hallmark of anaerobic metabolism, mainly reflecting an impaired condition of tissue hypoperfusion and hemodynamic instability (30). Elevated blood lactate levels are common and a lower clearance of serum lactate is robustly associated with adverse clinical outcomes in critically ill patients (31). Dai et al. have found that blood lactate can be used as a prognostic marker for ARDS patients receiving mechanical ventilation (21). However, due to the retrospective design of our study, causality could not be directly determined. This association warrants further in-depth research.

Some limitations in our study should be considered. First, patients in the MIMIC-IV database were recorded from 2008 to 2019. The care versions may have changed during this period, which might have affected results. Therefore, we have considered the admission period (before or after 2013) as an important confounder in the analysis. Second, due to a lack of relevant data, some subgroup analyses of interest couldn’t be performed, such as those by inflammatory status or fluid-management strategies. Third, our study is a retrospective design. The lack of a standardized criteria to perform CVP measurement for patients with or at risk for ARDS may limit the generalizability of these results. Therefore, a large cohort study and randomized controlled trials (RCT) are needed to validate our findings.

## Conclusion

In this retrospective analysis, we demonstrated that early use of CVP measurement during ICU stay could effectively decrease the 28-day mortality for patients with or at risk for ARDS. The beneficial effect of CVP measurement on 28-day mortality remain robust after the adjustment of confounding factors. Due to the retrospective non-randomized nature of our study, a causal relationship could not be ascertained. Further studies are required to determine the cause-and-effect relationships between CVP measurement and patient outcomes.

## Data Availability Statement

The raw data supporting the conclusions of this article will be made available by the authors, without undue reservation.

## Ethics Statement

The studies involving human participants were reviewed and approved by Ethics Committees of the Second Affiliated Hospital of Chongqing Medical University. Written informed consent for participation was not required for this study in accordance with the national legislation and the institutional requirements.

## Author Contributions

RT, JP, and DW: conception and design. RT and JP: data acquisition and data analysis. All authors: drafting and critically revising manuscript and final approval for publication.

## Conflict of Interest

The authors declare that the research was conducted in the absence of any commercial or financial relationships that could be construed as a potential conflict of interest.

## Publisher’s Note

All claims expressed in this article are solely those of the authors and do not necessarily represent those of their affiliated organizations, or those of the publisher, the editors and the reviewers. Any product that may be evaluated in this article, or claim that may be made by its manufacturer, is not guaranteed or endorsed by the publisher.
