# Supplementary material for: Central Venous Pressure Measurement Is Associated With Improved Outcomes in Patients With or at Risk for Acute Respiratory Distress Syndrome: An Analysis of the Medical Information Mart for Intensive Care IV Database
Source: Front Med (Lausanne). 2022 Mar 28;9:858838. doi: 10.3389/fmed.2022.858838 (PMC8995425; doi:10.3389/fmed.2022.858838)

Table S1. Percentage of missing data in variables of interest

Variables	Missing value(n=10198)	Percent (%)
Age	0	0%
Sex (Man, %)	0	0%
BMI (kg/m2)	2223	21.80%
Ethnicity	0	0%
Admission type	0	0%
Admission period	0	0%
first care unit	0	0%
Hypertension	0	0%
Coronary atherosclerosis	0	0%
Diabetes	0	0%
COPD	0	0%
Tumor	0	0%
Charlson score	0	0%
Pneumonia	0	0%
Sepsis	0	0%
Trauma	0	0%
Others	0	0%
APS III	0	0%
LODS	0	0%
OASIS	0	0%
Temperature (℃)	76	0.75%
HR (beats/min)	0	0%
RR (times/min)	0	0%
MAP (mmHg)	2367	23.21%
PaO2/FiO2 at diagnosis (mmHg)	0	0%
PEEP at diagnosis (cmH2O)	0	0%
WBC (k/uL)	3	0.03%
Hemoglobin (g/L)	3	0.03%
Platelet (k/uL)	3	0.03%
Bicarbonate (mEq/L)	2	0.02%
Bun (mg/dL)	2	0.02%
Creatinine (mg/dL)	2	0.02%
Lactate (mmol/L)	582	5.71%
Glucose (mg/dL)	3	0.03%
Sodium (mEq/L)	2	0.02%
potassium (mEq/L)	2	0.02%

Figure S1. Kaplan-Meier survival curves of 28-day mortality comparing CVP versus no CVP groups

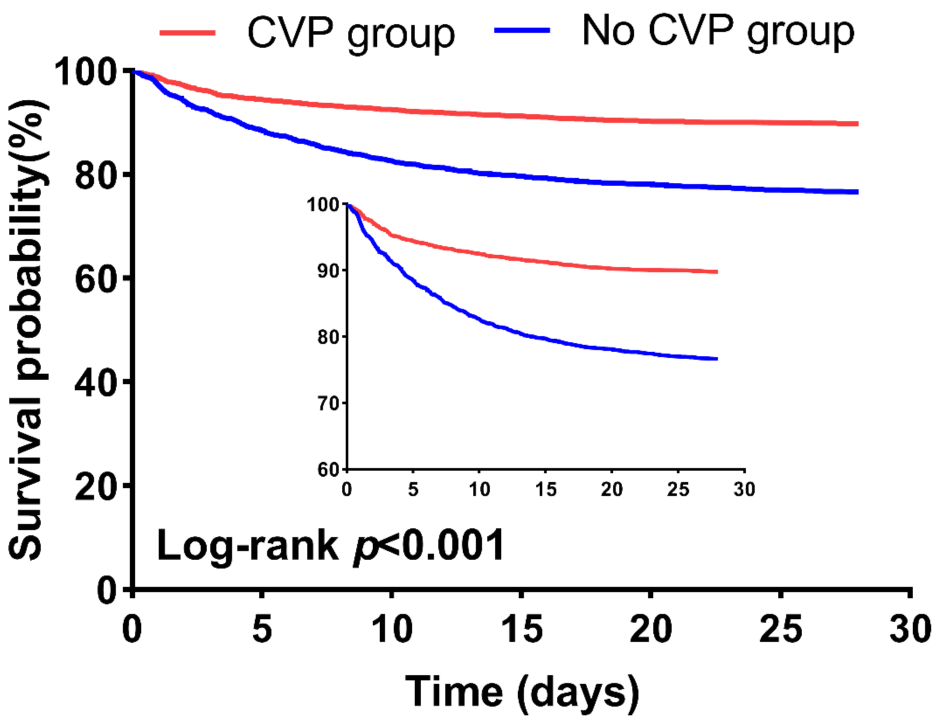

Supplement: Supplementary file 1 [file Data_Sheet_1.pdf]
